# Multifunctional Bacterial Cellulose Films Incorporating Kratom (*Mitragyna speciosa* Korth.) Leaf Extract as a Preliminary Antibacterial and Cytocompatible Wound Dressing Candidate

**DOI:** 10.3390/nano16150939

**Published:** 2026-07-30

**Authors:** Arnon Khamkeaw, Suwaphit Thaksin, Thitiwan Pechsiri, Phasuwit P. Phatchayawat, Suchada Sukrong, Suttinun Phongtamrug, Muenduen Phisalaphong

**Affiliations:** 1 Department of Biotechnology, Faculty of Applied Science, King Mongkut’s University of Technology North Bangkok, Bangkok 10800, Thailand; arnon.k@sci.kmutnb.ac.th; 2 Bio-Circular-Green-Economy Technology & Engineering Center (BCGeTEC), Department of Chemical Engineering, Faculty of Engineering, Chulalongkorn University, Bangkok 10330, Thailand; 6130590621@alumni.chula.ac.th (S.T.); thitiwanpechsiri@gmail.com (T.P.); 3 Department of Industrial Physics and Medical Instrumentation, Faculty of Applied Science, King Mongkut’s University of Technology North Bangkok, Bangkok 10800, Thailand; phasuwit.p@sci.kmutnb.ac.th; 4 Center of Excellence in DNA Barcoding of Thai Medicinal Plants, Department of Pharmacognosy and Pharmaceutical Botany, Faculty of Pharmaceutical Sciences, Chulalongkorn University, Bangkok 10330, Thailand; suchada.su@chula.ac.th; 5 Chulalongkorn School of Integrated Innovation, Chulalongkorn University, Bangkok 10330, Thailand; 6 Department of Industrial Chemistry, Faculty of Applied Science, King Mongkut’s University of Technology North Bangkok, Bangkok 10800, Thailand; suttinun.p@sci.kmutnb.ac.th

**Keywords:** bacterial cellulose, mitragynine, Kratom, *Mitragyna speciosa* Korth., entrapment, extraction

## Abstract

Leaves of *Mitragyna speciosa* Korth. (*M. speciosa*), commonly known as Kratom, are rich in alkaloids, with mitragynine as the main bioactive compound. This study presents a technique for the entrapment of ethanolic Kratom leaf extract within bacterial cellulose (BC) films and the subsequent controlled release of mitragynine. Kratom leaves were dried, ground, and extracted by maceration in 95% ethanol at 35 °C for 72 h. The extract was incorporated into the BC matrix via the immersion method, followed by air-drying at room temperature (~32 °C). BC’s highly porous structure facilitates sustained mitragynine absorption and entrapment within a tight nanofibrillar network. The mitragynine content in the BC films ranged from 9.1 to 23.3 mg/g, allowing for the evaluation of its effects on film properties and drug release performance. Release studies were conducted using Franz diffusion cells, with acetate buffer (pH 5.5) and phosphate buffer (pH 7.4) as receptor phases. Consistent with its higher solubility in acidic conditions, mitragynine showed greater release in acetate buffer, particularly within the first 0–12 h. The release profile depended on both mitragynine loading and time. The mitragynine-loaded BC films exhibited strong antimicrobial activity, achieving 100% reduction of *Staphylococcus aureus* and *Escherichia coli*. In vitro studies using L929 mouse fibroblast cells demonstrated that the films were noncytotoxic. The films also promoted proliferation and viability of normal human epidermal keratinocytes. Overall, the mitragynine-loaded BC films support skin cell growth and attachment and exhibit antibacterial, antioxidant, and anti-inflammatory properties, highlighting their potential as candidates for wound healing applications.

## 1. Introduction

*Mitragyna speciosa* Korth. (*M. speciosa*), known as Kratom in Thailand, is a plant belonging to the Rubiaceae family that is native to Southeast Asia [1]. *Mitragyna speciosa* contains many alkaloids, flavonoids, phenolics, and other bioactive components [2]. Mitragynine is the most abundant active alkaloid in *M. speciosa* and has various pharmacological properties. The mitragynine content of *M. speciosa* in Thailand has been reported to account for around 66% of the total alkaloids [3]. *Mitragyna speciosa* has been used in pharmacy and medicine for centuries in Southeast Asia [1,4]. *Mitragyna speciosa* extract is widely documented for its bioactive properties, including antinociceptive and analgesic effects [1], antimicrobial activity [5], antioxidant and anti-inflammatory activities [3,6], and cancer cell inhibition [7]. Mitragynine has been reported to potentially reduce pain signaling in inflammatory and neuropathic conditions [8]. In addition, due to its stimulant effects, it has been used to treat minor ailments and counter fatigue among manual laborers [2]. However, evidence from studies on high-dose and long-term use of mitragynine indicates that it may cause neuronal disorders [9]. Mitragynine exhibits a dose-dependent safety profile, in which low or subchronic doses are relatively safe, whereas high or chronic doses are associated with toxicity and adverse neurocognitive effects [6,10]. It has been reported that oral doses of total alkaloid extract of *M. speciosa* or mitragynine at 200 mg/kg can cause lethality in rats [11]. Therefore, improved methods for controlling the dosage and release of mitragynine are essential to enhance its safety, efficacy, and therapeutic potential.

Entrapment of bioactive compounds within organic materials is a widely used technique with potential applications in many fields, including the pharmaceutical and food industries [12]. Entrapment refers to the incorporation of bioactive substances into appropriate carriers to control their release and improve bioavailability, solubility, and stability [13]. Previous reports of transdermal delivery assessments demonstrate that Kratom (*M. speciosa*) ethanolic extract-loaded polymer films are noncytotoxic, physically stable, and cause no skin irritation, supporting biomaterial-based delivery of Kratom extract as a safe topical approach [14,15].

Among the various materials available, bacterial cellulose (BC) is one of the most promising for entrapment of bioactive compounds. BC is a cellulosic biomaterial that has emerged as a renewable material in recent years [16,17]. It is produced by *Acetobacter xylinum* using sugars and other carbohydrate substrates as carbon sources. BC has a nanoporous structure formed by nanocellulose fibers and exhibits unique properties, including high porosity, a high degree of crystallinity, large surface area for adsorption, high tensile strength, high water retention capacity, low solubility, high biocompatibility, and resistance to organic solvents [18,19,20]. BC is also recognized for its high purity, excellent mechanical properties, and ability to form 3D nanostructured networks suitable for drug loading and controlled, sustained release. In our previous work, we demonstrated the successful entrapment of curcumin [21] and an ethanolic extract of mangosteen peel [22,23] within a BC matrix. These results indicated that BC is effective for the controlled release of such active compounds. Based on these findings, incorporating bioactive products from herbal extracts into BC nanofiber networks could enable the development of multifunctional, biocompatible, and sustainable materials for biomedical applications, such as wound dressings and antimicrobial, anticancer, or antioxidant films. While previous reports illustrate active exploration of biomaterial matrices to deliver Kratom alkaloids like mitragynine, no published reports utilize BC to date [24,25].

In this study, BC was used as a biopolymer matrix for the entrapment and controlled release of mitragynine from Kratom extract. Extract suspensions with varying concentrations of mitragynine were absorbed into BC hydrogel. The mitragynine-loaded BC hydrogel was then air-dried at room temperature (approximately 32 °C) to form a stable film, converting weak hydrogen bonding among cellulose fibrils into stronger, irreversible hydrogen bonding to produce mitragynine-loaded BC film (BC-M). The absorption and release characteristics of mitragynine from BC were evaluated. The antimicrobial properties of BC-M were assessed against *Escherichia coli* and *Staphylococcus aureus*. Cytotoxicity was evaluated using L929 mouse fibroblast cells, and biocompatibility was assessed using normal human epidermal keratinocyte (NHEK) cells.

## 2. Materials and Methods

### 2.1. Preparation of Mitragyna speciosa Extract

Kratom leaves were purchased from a local market in Bangkok, Thailand, and were stored in a plastic container and kept in a 4 °C refrigerator. The experiments were conducted within one week of purchase. The Kratom leaves were washed with deionized water and dried in a hot air oven at 50 °C for 24 h. The application of 50 °C hot dry air is a highly effective, low-temperature method to rapidly remove surface moisture while both preventing the thermal degradation of sensitive bioactive compounds and ensuring thorough drying within 24 h. The extraction was performed via maceration. The dried pieces were ground into powder by a ball mill (Retsch PM100, Retsch GmbH, Haan-Gruiten, Germany), and then 25 g of the powder was soaked in 250 mL of 95% ethanol (purchased from Merck, Rahway, NJ, USA) in a 500 mL Erlenmeyer flask at 35 °C with gently shaking at 70 rpm using an incubator shaker (Innova 4330, New Brunswick Scientific, Edison, NJ, USA) for 72 h. This method was selected based on prior optimization to maximize mitragynine yield while preventing thermal degradation [14].

The obtained ethanolic mixture was then filtered through a filter cotton sheet and filter paper (Whatman No. 1). The filter cotton sheet acted as a coarse pre-filter to retain large particles of Kratom leaf powder, while the underlying filter paper (Whatman No. 1) captured the remaining fine powder. The filtered extracts were stored in closed brown glass bottles and kept in a 4 °C refrigerator. The Kratom leaves used in this study were from a single batch. The concentration of mitragynine in Kratom extract solution was examined by high-performance liquid chromatography (HPLC, Nexera LC-40 series, Shimadzu, Kyoto, Japan). The chromatographic system was performed on a C18 column (150 × 4.6 mm, 5.0 μm particle size) at a temperature of 25 °C, using a gradient program with solvent A (0.1% formic acid) and solvent B (acetonitrile) at a flow rate of 1.0 mL/min. The injection volume was 10 μL, and the chromatograms were observed at a wavelength of 254 nm. The HPLC chromatograms display a sharp peak of mitragynine at around 14.8 min from a standard sample of mitragynine and an ethanolic Kratom extract solution, as shown in Figure S1. The calibration curve to determine unknown sample concentrations is shown in Figure S2.

### 2.2. Preparation of BC Film Containing Kratom Extract

**Figure 1:**
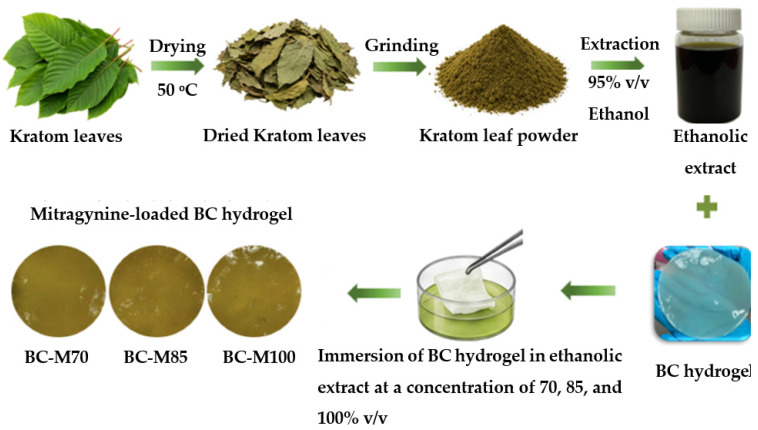
The preparation procedure of Kratom extract and BC film containing Kratom extract.

BC hydrogel films were biosynthesized according to the procedure previously reported [19]. The Kratom extract was diluted with 95% *v*/*v* ethanol at a concentration of the extract of 70, 85, and 100% *v*/*v*. BC hydrogel films with an average weight of 58.6 g (wet mass) were soaked in Kratom extract solutions of 100 mL at 30 °C for 24 h. The excess amount of extract on the film surface was then rinsed with 20% *v*/*v* of aqueous ethanol solution. Subsequently, the composite films were air-dried at room temperature (~32 °C) for 48 h and preserved in a sealed plastic bag. The composite films were prepared by immersing BC hydrogel films into the Kratom extract solution at a concentration of 70, 85, or 100% *v*/*v,* which were thereafter called BC-M70, BC-M85, and BC-M100, respectively. The preparation procedure for Kratom extract and BC film containing Kratom extract is shown in Figure 1.

### 2.3. Characterization

The bulk crystal structure was observed by X-ray diffraction (XRD) (SmartLab, RIGAKU, Tokyo, Japan). The surface functional groups were identified by Fourier transform infrared (FT-IR) (Spectrum 3TM, PerkinElmer, Shelton, CT, USA) spectroscopy. The mechanical properties were evaluated by a universal testing machine (UTM) (QC-506M1, Cometech, Taiwan). The thermal stability and mass loss were investigated by thermogravimetric analysis (TGA) (TGA/DSC1, Mettler Toledo, Greifensee, Switzerland). The surface area, porosity, and micro/meso-pore size distribution of samples were determined by nitrogen (N_2_) physisorption–desorption using a micromeriticsm chemisorb 2750 pulse (Micromeritics, Norcross, GA, USA). The morphology of the cells was observed using a scanning electron microscope (SEM) (Model of JSM-7610F, JEOL, Peabody, MA, USA).

### 2.4. Moisture Content

The samples were cut into square pieces of 2.0 cm^2^, and then the samples were weighed accurately. The samples were dried in a hot air oven at 70 °C until a fixed dry weight was achieved [26]. The weight of the sample at the beginning and the dry weight were recorded, and the percentage of moisture content was calculated as follows:

$$Moisture\;content\left(\%\right)=\frac{W_{0}-W_{1}}{W_{0}}\times 100$$

where W_0_ is the weight of sample at the beginning, and W_1_ is the dry weight of the sample.

### 2.5. Water Solubility

The dried square samples of 2.0 cm^2^ were precisely weighed and recorded. The samples were soaked in 100 mL of DI water under stirring at 180 rpm for 24 h at ambient temperature [27]. After 24 h, the remaining portions of the sample were filtered and were then dried in a hot air oven at 70 °C until a constant weight was achieved. The percentage of water solubility was determined as follows:

$$Water\;solubility\left(\%\right)=\frac{W_{i}-W_{f}}{W_{i}}\times 100$$

where W_i_ is the initial dried weight, and W_f_ is the final dried weight of remaining samples.

### 2.6. Water Holding Capacity

The dried samples of 2 cm^2^ were immersed in DI water for 6 h. The samples were then taken out of the container. The samples were dried at 70 °C for 24 h in order to completely remove water [28]. The water holding capacity was determined as follows:

$$Water\;holding\;capacity\left(\%\right)=\frac{Mass\;of\;water\;removed\;during\;drying\;(g)}{Dry\;weight\;of\;sample\;(g)}\times 100$$

### 2.7. Antimicrobial Testing

The modified Japan industrial standard test JIS Z 2801:2006 for testing the antibacterial activity of films was applied for the study of antimicrobial properties [29,30]. *Staphylococcus aureus* (Gram-positive) and *Escherichia coli* (Gram-negative) bacteria were grown in a standardized culture medium. The sterilized sample films of 5 × 5 cm^2^ squares were placed inside covered Petri dishes and inoculated with 0.4 mL of a bacterial suspension. After incubation at 37 °C for 1 h, the samples were shaken thoroughly at 200 rpm for 1 min in the presence of 10 mL of pH 7.4 phosphate-buffered saline (PBS). Subsequently, the PBS solution containing the cells was cultured on agar plates and incubated at 37 °C for another 24 h. Then, the number of viable cells was estimated indirectly using the plate counting method [31] and expressed in CFU/mL and log CFU/mL. The percentage of microbial reduction was determined as follows:

(1) $$\mathrm{Reduction}\;(\%)\;=\frac{\left(C_{i}\;-{\;C}_{f}\right)}{C_{i}}\;\times \;100$$

where C_i_ is the concentration of bacteria (CFU/mL) before treatment at 0 h, and C_f_ is the bacteria concentration (CFU/mL) after treatment at 24 h.

### 2.8. Release Study

The release profile of mitragynine from BC films was investigated. The dried film measuring 35 mm in diameter was placed in modified Franz diffusion cells [21,23]. Physiological phosphate-buffered saline (PBS) at pH 7.4 and acetate buffer at pH 5.5, along with 0.5% *v*/*v* Tween 80 and 3% *v*/*v* methanol, were used as the release medium. The receptor chamber was fully filled with 11.5 mL of release medium. The release experiment was performed at 37 °C with constant stirring using a Teflon-coated magnetic stir bead for 48 h. During the release testing, 0.3 mL of sample was taken from the receptor through a sampling port, and an equal amount of the fresh buffer was immediately refilled. The concentration of released mitragynine was then analyzed by HPLC (Nexera LC-40 series, Shimadzu, Japan).

### 2.9. Cytotoxicity Assay

The cytotoxicity of BC films containing ethanolic extract of Kratom (BC, BC-M70, BC-M85, and BC-M100 films) was evaluated using L929 cells using the indirect extract assay. L929 cells were cultured in Dulbecco’s Modified Eagle Medium (DMEM) (Thermo Fisher Scientific, Waltham, MA, USA) supplemented with 10% fetal bovine serum (FBS) (Gibco, Grand Island, NY, USA) prior to the assay. The film samples were sterilized by autoclaving at 121 °C for 15 min and then immersed in DMEM and incubated at 37 °C for 24 h to prepare the sample extract. The resulting cell culture medium was used to assess the effect on L929 cell viability. L929 cells were cultured in 96-well plates at a density of 1 × 10^4^ cells/well and incubated at 37 °C under 5% CO_2_ for 24 h. Afterwards, the cell culture medium in each well was changed to either the sample extract, negative control medium, or positive control medium. The negative control group used DMEM cell culture medium without any cytotoxic substances, while the positive control group used DMEM cell culture medium supplemented with 10% (*v*/*v*) dimethyl sulfoxide or DMSO. L929 cells were then incubated at 37 °C under 5% CO_2_ for 24 h. L929 cells viability was assessed using the PrestoBlue™ cell viability reagent kit (Thermo Fisher Scientific, Waltham, MA, USA) according to the manufacturer’s instructions. Each well was filled with 100 µL of DMEM containing 10% (*v*/*v*) PrestoBlue™, and the plates were covered with aluminum foil to protect them from light and incubated at 37 °C for 1 h. After incubation, absorbance measurements at wavelengths of 570 and 600 nm were taken using a Multiskan GO spectrophotometer (Thermo Fisher Scientific, Waltham, MA, USA).

### 2.10. Biocompatibility Testing

Biocompatibility testing of BC, BC-M70, BC-M85, and BC-M100 films was performed using normal human epidermal keratinocyte (NHEK) cells (C-12006, PromoCell, Heidelberg, Germany). NHEK cells were cultured in keratinocyte growth medium (Gibco, Grand Island, NY, USA) supplemented with 2.44% supplement mix and 0.06 mM CaCl_2_ in 75 cm^2^ cell culture flasks (Corning, New York, NY, USA) and incubated at 37 °C under 5% CO_2_, changing the culture medium every 3–4 days. When cell density reached approximately 80%, cell transfer was performed using TrypLE™ Express (Gibco, Grand Island, NY, USA) for direct film culture. The film samples were cut into 1 cm diameter circular discs, and NHEK cells were cultured onto each film disc at a density of 5 × 10^4^ cells/film, using cover slides as a control group. NHEK cells culture samples were then cultured in 24-well plates (Corning, Grand Island, NY, USA) using keratinocyte growth medium supplemented with 2.44% supplement mix and 0.06 mM CaCl_2_ under standard culture conditions, with the culture medium changed every 3–4 days. NHEK cells proliferation on the film samples was assessed after 1, 3, and 7 days of culture using the PrestoBlue™ cell viability assay. After the specified time, the NHEK cell-seeded films were gently washed with phosphate-buffered saline (PBS), and NHEK cells were immobilized with 2.5% glutaraldehyde. The NHEK cell-immobilized film samples were then dried using progressively concentrated ethanol solutions and subsequently dried using a Leica EM CPD300 critical point dryer (Leica Microsystems, Wien, Austria). The NHEK cell-immobilized film samples were coated with gold using the sputter coating method and examined using the scanning electron microscope (SEM) model JEOL JSM-IT500HR (JEOL, Tokyo, Japan) to evaluate the morphology, adhesion, and distribution of NHEK cells on the film surface.

### 2.11. Statistical Analysis

The quantitative data are presented in the form of the mean ± standard deviation. Statistical analysis was performed using GraphPad Prism version 10.1.0. Differences between experimental groups were analyzed using two-way analysis of variance (two-way ANOVA), followed by multiple comparison tests using Tukey’s honestly significant difference test. The statistical significance level was set at *p* < 0.05.

## 3. Results and Discussion

### 3.1. Property of BC Film

The properties of the dried BC film are shown in Table 1. The dried BC film has a thickness of 0.1 mm and exhibits high mechanical properties, with a tensile strength of 100 MPa and an elongation of 1.5%. The surface area and pore size were 115.3 m^2^/g and 21.7 nm, respectively. The BC had high water retention capacity and was highly water-resistant, with water holding capacity of 20.5 g/g, water solubility of 0%, and moisture content of 8.0%. These unique properties of BC originate from its three-dimensional (3D) network structure of nanocellulose fibers with high purity [29,32].

### 3.2. Adsorption of Mitragynine into BC Films

The amount of mitragynine in Kratom extract was determined by HPLC. The concentrations of mitragynine in the extracts BC-M70, BC-M85, and BC-M100 were 148.6, 174.9, and 205.1 μg/mL, respectively. Adsorption was carried out by immersing never-dried BC in Kratom extract at 30 °C for 24 h. As the concentration of Kratom extract increased, more mitragynine molecules were absorbed into the BC matrix. The actual amounts of mitragynine absorbed into BC-M70, BC-M85, and BC-M100 films were 9.1, 15.8, and 23.3 mg/g, respectively. These concentrations (9.1–23.3 mg/g) are comparable to the typical range found in naturally growing Kratom leaves in Thailand (7.5–26.6 mg/g of dry leaf weight) [33]. BC provides a highly porous structure that facilitates sustained mitragynine absorption, allowing the molecules to penetrate the loose matrix of the never-dried BC films during immersion. Subsequently, mitragynine becomes entrapped within a tighter BC nanofibrillar network during the drying step. Due to its highly porous 3D nanofibrillar structure, BC offers high drug loading capacity, excellent water retention of 20.5 g/g, and strong mechanical properties, with tensile strength of ~100 MPa and elongation of 1.5%. Therefore, BC shows potential as a carrier with high absorption capacity of mitragynine.

### 3.3. XRD Analysis

**Figure 2:**
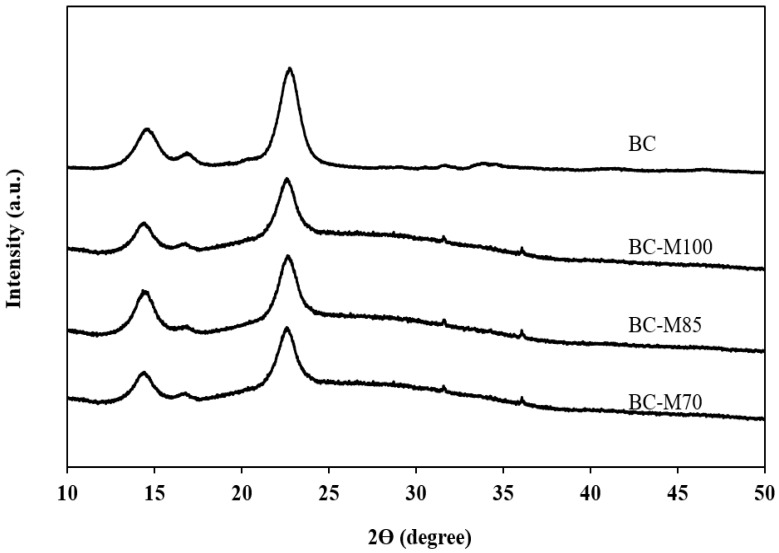
The XRD pattern of the films.

The XRD profiles of BC, BC-M70, BC-M85, and BC-M100 films are shown in Figure 2. The XRD peaks at 14.7°, 16.8°, and 22.8°, which corresponds to the main planes of the cellulose crystal (100; 010; 110) in BC structure [34,35,36]. The XRD profiles of BC, BC-M70, BC-M85, and BC-M100 films showed peaks that were located similarly to those of BC. It indicated that the incorporation of Kratom extract into the BC matrix exhibits no significant effect on the structural properties of BC. Therefore, the structure integrity and strength of the BC were maintained after the incorporation of Kratom extract.

### 3.4. FTIR Analysis

**Figure 3:**
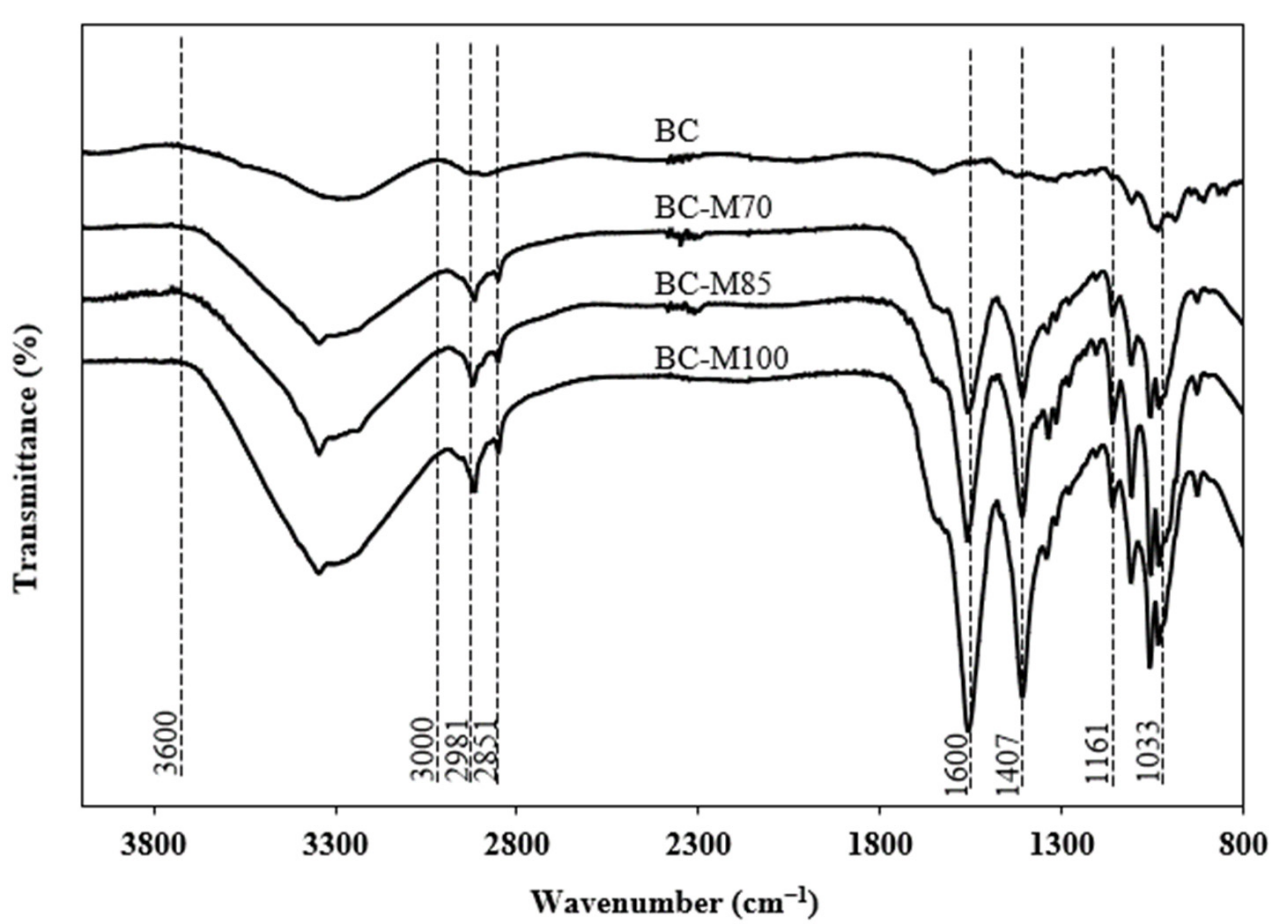
The FTIR pattern of the films.

The chemical surface functional groups of the BC, BC-M70, BC-M85, and BC-M100 are shown in Figure 3. The FTIR spectra of all samples show a broad band at around 3600–3000 cm^−1^, which is attributed to the O–H stretching vibration of the hydroxyl group. The presence of C–H stretching was also found at 2918 and 2851 cm^−1^. The broad absorption band at around 1161–1033 cm^−1^ can be associated with C–O–C asymmetric stretching of cellulose [37]. In the BC-M70, BC-M85, and BC-M100 films with Kratom leaf extract loading, the characteristic absorption peaks were identified at specific wavenumbers of ~1600, 1407, and 1033 cm^−1^, which correspond to C=O, C=C, and C–O vibrations in the mitragynine structure, respectively [38]. These wavenumbers align with the functional groups of the major Kratom alkaloid (mitragynine). The increase in intensity of these peaks follows the concentration of loading Kratom leaf extract for BC-M70, BC-M85, and BC-M100 films. The observed FTIR patterns therefore could indicate the successful preparation of BC incorporating Kratom leaf extract films.

### 3.5. Thermal Stability Study

**Figure 4:**
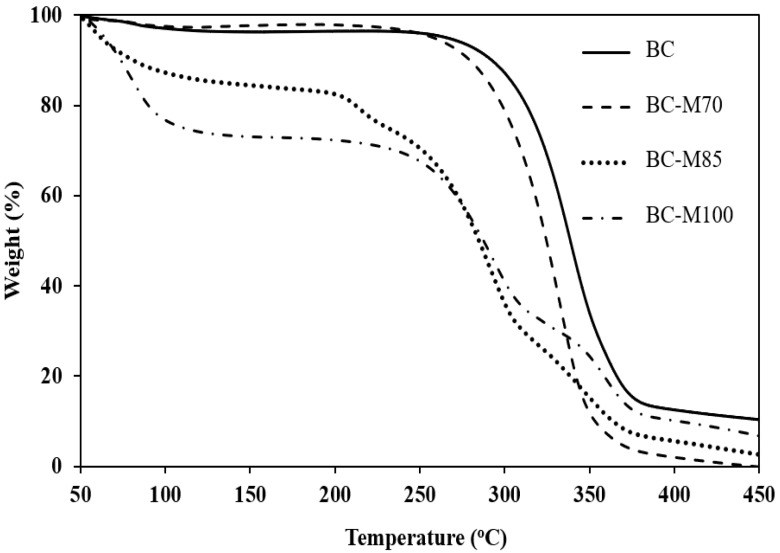
The TGA thermograms of the films.

The mass loss profile during thermal gravimetric analysis of BC, BC-M70, BC-M85, and BC-M100 is shown in Figure 4. The profiles showed that the trends of the TGA patterns of all composite films are similar. The initial mass loss at temperatures around 50 °C up to 110 °C could be described as the elimination of moisture in the film structure, which was approximately 5% of the initial weight. However, BC-M70, BC-M85, and BC-M100 films exhibit a higher mass loss than the BC film in the initial stage. This could be attributed to the partial degradation of Kratom extract. BC, BC-M70, BC-M85, and BC-M100 showed the structure decomposition temperature range between 250 to 350 °C. The TGA profiles indicate that the BC-M70, BC-M85, and BC-M100 films could be used for applications at temperatures up to approximately 250 °C.

### 3.6. Release Assay

**Figure 5:**
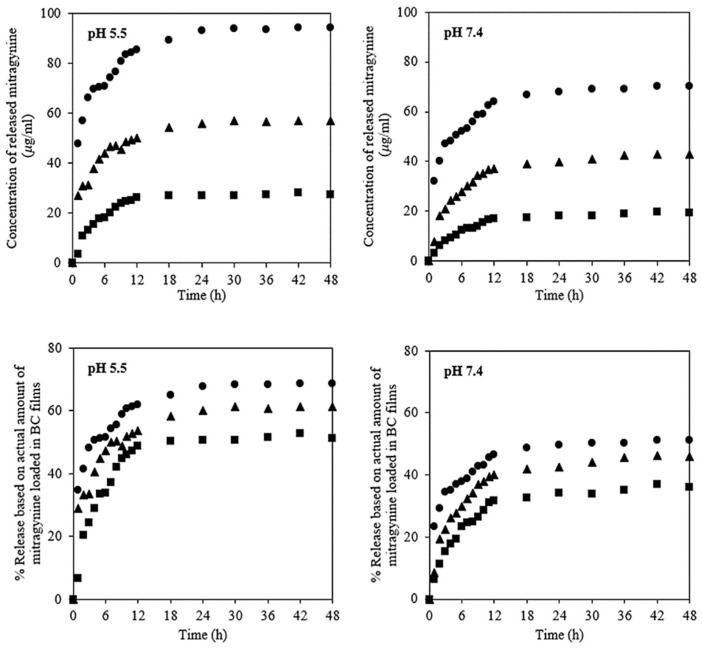
Release profiles of mitragynine from BC-M70 (◼), BC-M85 (▲), and BC-M100 (●) into buffer solutions (acetate buffer at pH 5.5 (**left**) and PBS buffer at pH 7.4 (**right**)): concentration of released mitragynine (**top**) and percentage of release based on actual amount of mitragynine loaded in BC films (**bottom**).

The concentration of released mitragynine and the percentage of release (Figure 5) increased with the amount of mitragynine absorbed into the BC films, following the order BC-M100 > BC-M85 > BC-M70. Release also increased with contact time. All samples exhibited a high release rate during the initial 0–12 h, which then gradually decreased until the process was completed within 48 h. After 24 h, the concentrations of released mitragynine from BC-M70, BC-M85, and BC-M100 into acetate buffer (pH 5.5) were 27.1, 55.8, and 92.9 µg/mL, respectively, whereas those in PBS buffer (pH 7.4) were 18.2, 39.6, and 68.1 µg/mL. The high release rate at the initial stage can be attributed to a higher mitragynine concentration gradient, which promotes rapid mass transport of mitragynine molecules from the BC matrix. The concentrations of mitragynine released into acetate buffer (pH 5.5) were higher than those in PBS buffer (pH 7.4) for all samples, likely because mitragynine is more soluble in acidic solutions than in neutral or basic conditions [33,39]. After 48 h, the release percentages of BC-M70, BC-M85, and BC-M100 in acetate buffer were 51.3%, 61.3%, and 68.6%, respectively, while those in PBS buffer were 36.1%, 46.0%, and 51.1%. The results indicate that mitragynine remains relatively stable in both acetate buffer (pH 5.5) and PBS buffer (pH 7.4). In previous work, Ramanathan et al. (2015) reported that mitragynine is highly stable in basic aqueous solutions (pH 7–9) but may degrade in acidic solutions at pH 4.0 [40]. Overall, these results further support the potential of BC as a suitable matrix for the absorption and controlled release of mitragynine.

### 3.7. Antimicrobial Activity

**Figure 6:**
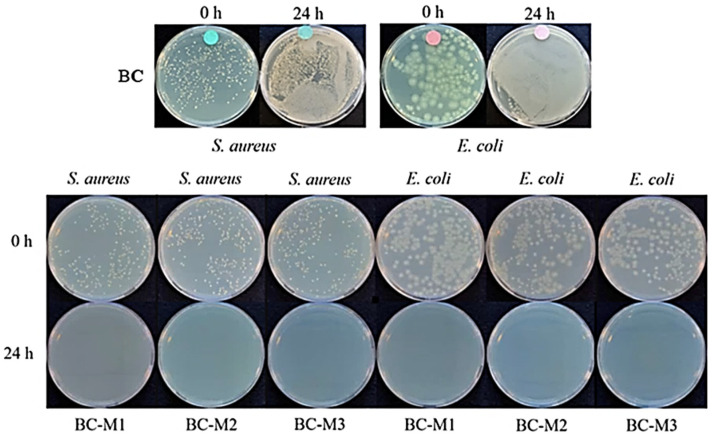
Antimicrobial properties of BC (control) (**top**) and BC-loaded Kratom extract (**bottom**).

The antimicrobial activities of BC-M70, BC-M85, and BC-M100 were evaluated using *S. aureus* and *E. coli* as models of Gram-positive and Gram-negative bacteria, respectively, as shown in Table 2 and Figure 6. After 24 h of incubation, no visible bacterial growth of either *S. aureus* or *E. coli* was observed on Petri dishes treated with BC-M70, BC-M85, and BC-M100. This indicates that these films exhibit strong antibacterial activity, achieving 100% cell reduction, whereas the BC film alone showed no antibacterial activity. These results suggest that Kratom extract released from the BC-M films could effectively kill the bacteria, leading to the absence of bacterial growth. This finding is consistent with earlier studies reporting that crude extracts of Kratom leaves are rich in mitragynine, a bioactive alkaloid recognized for its antimicrobial properties [5]. Salim et al. (2022) also reported that antimicrobial activity increased with increasing concentrations of Kratom extract (25–100%), with no bacterial growth observed at high concentrations [41].

### 3.8. Cytotoxicity

**Figure 7:**
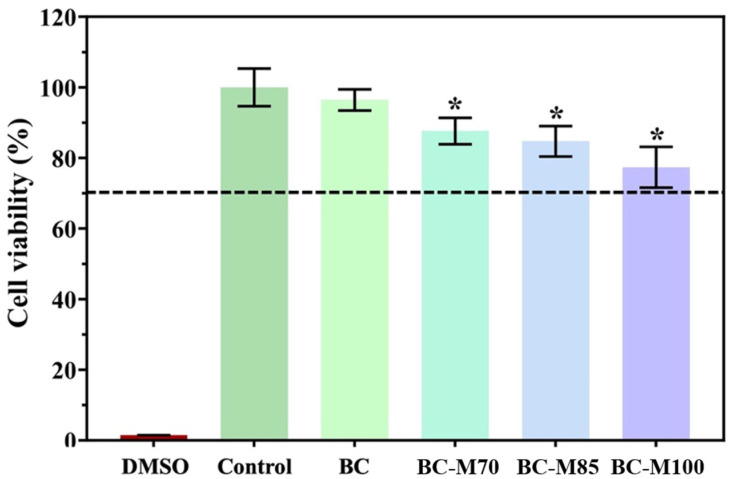
Cytotoxicity evaluation of films (BC, BC-M70, BC-M85, and BC-M100) using L929 cell line. The symbol * indicates a statistically significant difference (*p* < 0.05) versus Control.

The cytotoxic potential of BC films containing ethanolic extract of Kratom was evaluated using L929 mouse fibroblast cells (Figure 7). The BC film alone showed no significant effect on the viability of L929 cells. BC films incorporating Kratom extract (BC-M70, BC-M85, and BC-M100) exhibited a concentration-dependent decrease in cell viability. However, at the concentration range used in this study, cell viability remained above 75% in all extract-loaded groups. According to international standards such as ISO 10993-5, a reduction in cell viability of <30% (i.e., viability of >70%) compared with an untreated control is considered noncytotoxic. Therefore, these results indicate that the Kratom extract-loaded BC films prepared in this study are noncytotoxic at the tested concentrations. This is consistent with a broader body of literature reporting that Kratom/mitragynine display concentration-dependent cytocompatibility across multiple cell types, including L929, HaCaT, and NIH3T3 fibroblast/keratinocyte lines, with cytotoxicity typically emerging only above the low mg/mL range (Table S1) [24,25,42]. Previous studies have reported that mitragynine at 50 μg/mL significantly reduced the viability of C6 rat glioma (CCL107), SH-SY5Y human neuroblastoma (CRL2266), and HT22 immortalized mouse hippocampal neuronal (SCC129) cell lines [43]. Mitragynine at 1–25 µM has also been shown to induce dose-dependent increases in both mRNA and protein expression, along with enzyme activity, in human liver cells [44,45]. In particular, when administered orally at high concentrations, Kratom extract has been associated with cytotoxicity and potential liver and kidney damage. By contrast, transdermal delivery of Kratom extract via polymer film formulations offers a promising approach for controlled release of active compounds and has been reported as a safer, non-toxic alternative for potential therapeutic use. Studies on transdermal patches and films composed of agar, pectin, sodium alginate, and polyvinyl alcohol containing Kratom extract [15] have shown these systems to be generally noncytotoxic and biocompatible at low, controlled concentrations. Additionally, no signs of irritation were observed in rabbit skin screening tests, suggesting suitability for topical application [44].

### 3.9. Biocompatibility

#### 3.9.1. Cell Proliferation

**Figure 8:**
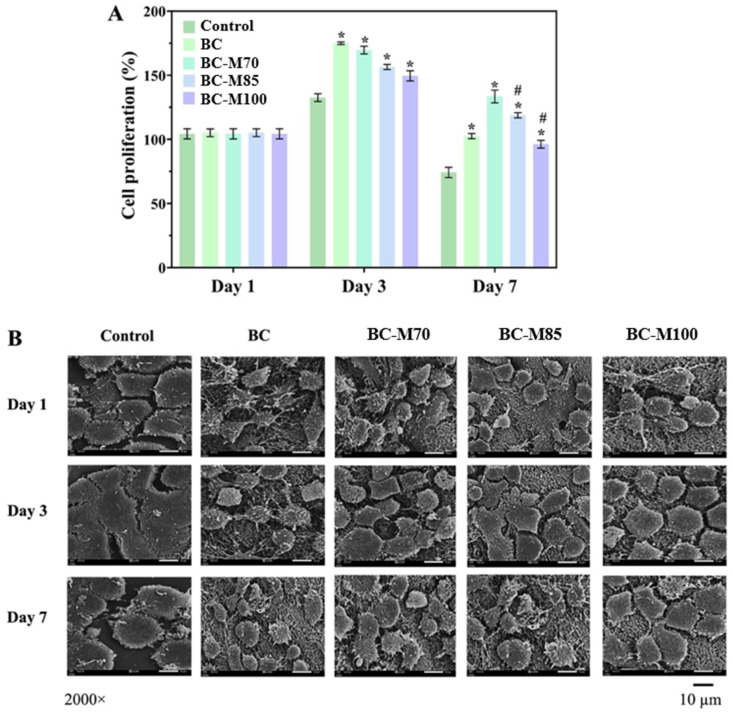
Biocompatibility evaluation of films (BC, BC-M70, BC-M85, and BC-M100) using normal human epidermal keratinocytes (NHEKs): (**A**) relative proliferation at Days 1, 3, and 7. (Data are presented as % relative to Day 1 control. * *p* < 0.05 vs. control at the same time point; # *p* < 0.05 vs. Day 1 of the same sample). (**B**) SEM images showing cell attachment and morphology on different film surfaces at Days 1, 3, and 7.

The biocompatibility of the films was further assessed by evaluating normal human epidermal keratinocytes (NHEKs) proliferation over a period of 7 days (Figure 8). As shown in Figure 8A, no significant differences in proliferation were observed between groups on Day 1, indicating no significantly direct cytotoxic effects on NHEKs. On Day 3, all extract-loaded films (BC-M70, BC-M85, and BC-M100), as well as the BC film, exhibited significantly greater cell proliferation than the control (*p* < 0.05). Among these, the BC film showed the highest proliferation, followed by BC-M70, BC-M85, and BC-M100, respectively. This suggests a positive effect of BC films on cell growth stimulation. BC hydrogel is widely recognized for its high biocompatibility, high porosity and surface area, excellent water holding capacity, and suitable mechanical strength, all of which support cell proliferation. On Day 7, cell proliferation decreased in all treatment groups, possibly due to depletion of nutrients and accumulation of waste products during prolonged incubation. However, BC-M70 and BC-M85 films showed a significantly lower rate of cell loss compared with the control and BC film. This result suggests that at low to moderate concentrations, the ethanolic extract of Kratom may help protect against cell damage. Mitragynine has been reported to exhibit antioxidant properties by reducing oxidative stress and acting as a radical scavenger, which could contribute to protection against cellular damage [45]. This protective effect is consistent with reports of mitragynine and Kratom extract scavenging DPPH, ABTS, and ROS radicals in cell-based systems, with effective concentrations generally in the low µg/mL–mg/mL range (Table S2) [24,42].

Kratom extract has been widely reported to have potential therapeutic benefits. Mitragynine acts on μ-opioid and α-adrenergic receptors and has shown potential in reducing chemotherapy-induced neuropathic pain [46]. Kratom extract has also been reported to inhibit inflammatory mediators and cytokine expression [14,47]. The anti-inflammatory activity of Kratom extract is associated with mitragynine, which inhibits prostaglandin E2 formation through the suppression of COX-2 expression [47,48]. Beyond COX-2/PGE2 suppression, more recent studies further demonstrate that Kratom alkaloid extracts and methanolic extracts down-regulate pro-inflammatory mediators including TNF-α, IL-6, IL-1β, iNOS, and NF-κB(p65) nuclear translocation, with associated modulation of the ERK/JNK (MAPK) signaling pathway and, in some studies, increased anti-inflammatory IL-10 production via TLR-4 inhibition (Table S3) [42,49,50]. Furthermore, Kratom leaf extract and its ethyl acetate/dichloromethane fractions have been directly evaluated for wound healing-relevant cellular functions, including in vitro scratch/migration assays on 3T3 fibroblasts, tube formation (angiogenesis) assays on HUVECs, and cytocompatibility below 50 µg/mL, supporting the biological plausibility of Kratom-loaded biomaterials as wound healing candidates (Table S4) [51]. Previous studies have reported that ethanolic extracts of Kratom exhibit relatively low cytotoxicity across various human cell lines. For example, these extracts have shown low cytotoxic effects on human skin cells, with half-maximal inhibitory concentrations (IC_50_) ranging from 1.4 to 2.5 mg/mL [52]. The IC_50_ values for cell proliferation of fibroblasts, melanocytes, macrophages, and keratinocytes were reported to be approximately 2.46, 1.99, 1.40, and 1.29 mg/mL, respectively [47]. Additionally, ethanolic extracts showed IC_50_ values greater than 500 µg/mL in kidney (HEK-293) and liver (HeLa Chang) cell lines [47]. Similarly, nanoparticle-based Kratom formulations tested on HaCaT keratinocytes showed IC50 values in the 0.7–1.3 mg/mL range depending on variety and formulation, further supporting the cytocompatibility window relevant to topical/wound dressing applications (Table S4) [24]. Thus, while mitragynine exhibits potential therapeutic benefits at low concentrations, it may become inhibitory or cytotoxic at higher concentrations. Therefore, controlling the release of mitragynine at an appropriate concentration level is important to avoid toxicity and other adverse effects.

#### 3.9.2. Cell Morphology

SEM imaging was used to visualize NHEK morphology and attachment on the surfaces of BC, BC-M70, BC-M85, and BC-M100 films at Days 1, 3, and 7 (Figure 8B). On Day 1, cells on the control (cover glass) appeared rounded with some spreading. In comparison, the extract-loaded BC films showed good cell attachment with a greater degree of spreading than the BC film, indicating favorable surface interactions. By Day 3, cells on the BC-M85 and BC-M100 films exhibited more pronounced spreading and the formation of interconnected networks, while moderate cellular extensions were observed on BC-M70 films. By Day 7, all groups showed reduced cell coverage on the surface. These morphological observations are consistent with the proliferation assay results (Figure 8A). Overall, BC films loaded with ethanolic Kratom extract demonstrate good biocompatibility with NHEK cells. These morphological findings are broadly consistent with the cell attachment and spreading behavior reported for Kratom/mitragynine-based biomaterial platforms, including polymer film patches, nanoparticle-loaded gels, nanostructured lipid carriers, and chitosan microencapsulation systems (Table S1) [24,25,42,43], none of which, to our knowledge, have previously used BC as the delivery matrix, underlining the novelty of the present BC–Kratom film system. The films’ functions as supporters for cell adhesion and proliferation and their biocompatibility, combined with strong antibacterial activity as well as antioxidant and anti-inflammatory properties, make them promising candidates for wound healing applications. A comprehensive summary of evidence supporting the wound healing potential, antioxidant activity, anti-inflammatory activity, and cytocompatibility of Kratom/mitragynine is provided in Tables S1–S4, respectively.

## 4. Conclusions

Entrapment of mitragynine in BC films was successfully achieved. The mitragynine-loaded BC films of BC-M70, BC-M85, and BC-M100 contained 9.1, 15.8, and 23.3 mg/g of mitragynine in dry film, respectively. The BC-M films exhibited strong antimicrobial activity against *E. coli* and *S. aureus*, with 100% reduction in bacterial viability. Mitragynine remained stable in both acidic buffer (pH 5.5) and neutral buffer (pH 7.4). The release of mitragynine from BC films into acidic and neutral buffers reached 48.7–62.1% and 31.6–46.7%, respectively, within the first 0–12 h. These findings indicate that BC has strong potential as a carrier with high absorption capacity and controlled release capability for mitragynine. For potential application as a topical wound healing patch, the cytotoxicity and biocompatibility of the modified BC films were evaluated. The results demonstrate that Kratom extract-loaded BC films are noncytotoxic to L929 mouse fibroblast cells and exhibit good biocompatibility with dermal cells (NHEKs). The combination of biocompatibility, antibacterial activity, antioxidant and anti-inflammatory properties, and moisture retention capacity suggests that BC-M films have strong potential as a preliminary antibacterial and cytocompatible wound dressing candidate. However, further studies, especially regarding their long-term safety, are required for advanced and bio-active regenerative therapies in skin wound healing.

## Figures and Tables

**Table 1:** The property of BC film.

Property	Value
Thickness (mm)	0.1
Tensile strength (MPa)	100
Elongation (%)	1.5
BET surface area (m^2^/g)	115.3
Pore size (nm)	21.7
Moisture content (%)	8.0
Water holding capacity (g/g)	20.5
Water solubility (%)	0

**Table 2:** The number of microbial cells at 0 and 24 h.

Sample	*S. aureus*			*E. coli*		
	Log CFU/mL		% Reduction	Log CFU/mL		% Reduction
	0 h	24 h		0 h	24 h	
BC	6.19 ± 0.05	>LOD	-	6.28 ± 0.03	>LOD	-
BC-M70	6.19 ± 0.04	<LOD	100	6.27 ± 0.04	<LOD	100
BC-M85	6.20 ± 0.03	<LOD	100	6.27 ± 0.01	<LOD	100
BC-M100	6.20 ± 0.03	<LOD	100	6.28 ± 0.01	<LOD	100

>LOD (Greater than the Limit of Detection); <LOD (Below Limit of Detection).

## Data Availability

The original contributions presented in this study are included in the article/Supplementary Materials. Further inquiries can be directed to the corresponding author.

## Supplementary Materials

The following supporting information can be downloaded at https://www.mdpi.com/article/10.3390/nano16150939/s1, Figure S1. A high-performance liquid chromatography (HPLC) chromatogram displays a sharp peak of mitragynine at around 15 min from a standard sample (A) and from an ethanolic Kratom extract solution (B); Figure S2. A standard curve of concentration (y-axis) vs. peak area (x-axis) made from standard samples of mitragynine for a calibration method to quantify unknown samples; Table S1. Biological compatibility and cytocompatibility of Kratom/mitragynine-based systems; Table S2. Antioxidant activities reported for Kratom; Table S3. Anti-inflammatory activities reported for Kratom/mitragynine; Table S4. Evidence supporting wound healing potential of Kratom and mitragynine.
