# Supplementary material for: Multifunctional Bacterial Cellulose Films Incorporating Kratom (Mitragyna speciosa Korth.) Leaf Extract as a Preliminary Antibacterial and Cytocompatible Wound Dressing Candidate
Source: Nanomaterials (Basel). 2026 Jul 30;16(15):939. doi: 10.3390/nano16150939 (PMC13468205; doi:10.3390/nano16150939)
Supplement: Supplementary file 1 [file nanomaterials-16-00939-s001.zip › nanomaterials-4429831-supplementary.pdf]

Supporting Information

Multifunctional Bacterial Cellulose Films Incorporating Kratom (*Mitragyna speciosa* Korth.) Leaf Extract as a Preliminary Antibacterial and Cytocompatible Wound Dressing Candidate

Arnon Khamkeaw [1], Suwaphit Thaksin [2], Thitiwan Pechsiri [2], Phasuwit P. Phatchayawat [3], Suchada Sukrong [4,5], Suttinun Phongtamrug [6] and Muenduen Phisalaphong [2,*]

[1] Department of Biotechnology, Faculty of Applied Science, King Mongkut's University of Technology North Bangkok, Bangkok 10800, Thailand; arnon.k@sci.kmutnb.ac.th
[2] Bio-Circular-Green-Economy Technology & Engineering Center (BCGeTEC), Department of Chemical Engineering, Faculty of Engineering, Chulalongkorn University, Bangkok 10330, Thailand; 6130590621@alumni.chula.ac.th (S.T.); thitiwanpechsiri@gmail.com (T.P.)
[3] Department of Industrial Physics and Medical Instrumentation, Faculty of Applied Science, King Mongkut's University of Technology North Bangkok, Bangkok 10800, Thailand; phasuwit.p@sci.kmutnb.ac.th
[4] Center of Excellence in DNA Barcoding of Thai Medicinal Plants, Department of Pharmacognosy and Pharmaceutical Botany, Faculty of Pharmaceutical Sciences, Chulalongkorn University, Bangkok 10330, Thailand; suchada.su@chula.ac.th
[5] Chulalongkorn School of Integrated Innovation, Chulalongkorn University, Bangkok 10330, Thailand
[6] Department of Industrial Chemistry, Faculty of Applied Science, King Mongkut's University of Technology North Bangkok, Bangkok 10800, Thailand; suttinun.p@sci.kmutnb.ac.th
* Correspondence: muenduen.p@chula.ac.th

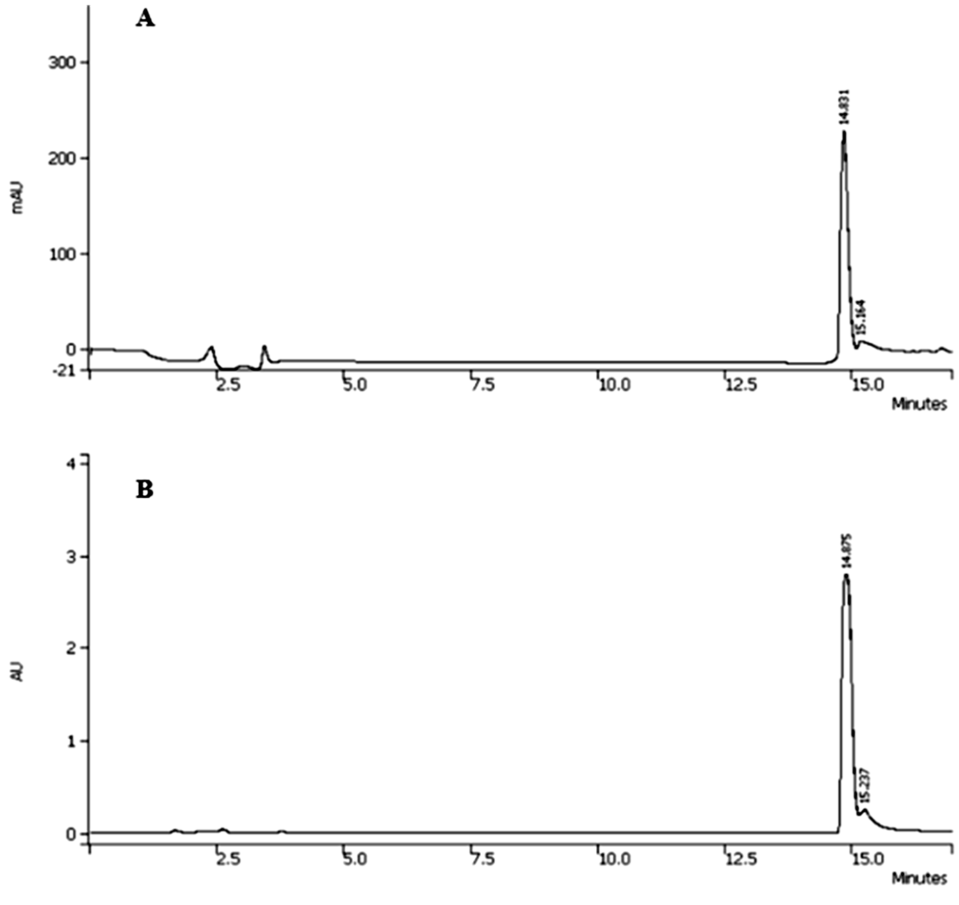

Figure S1. A High-Performance Liquid Chromatography (HPLC) chromatogram displays a sharp peak of mitragynine at around 14.8 minutes from a standard sample (A) and from an ethanolic Kratom extract solution (B).

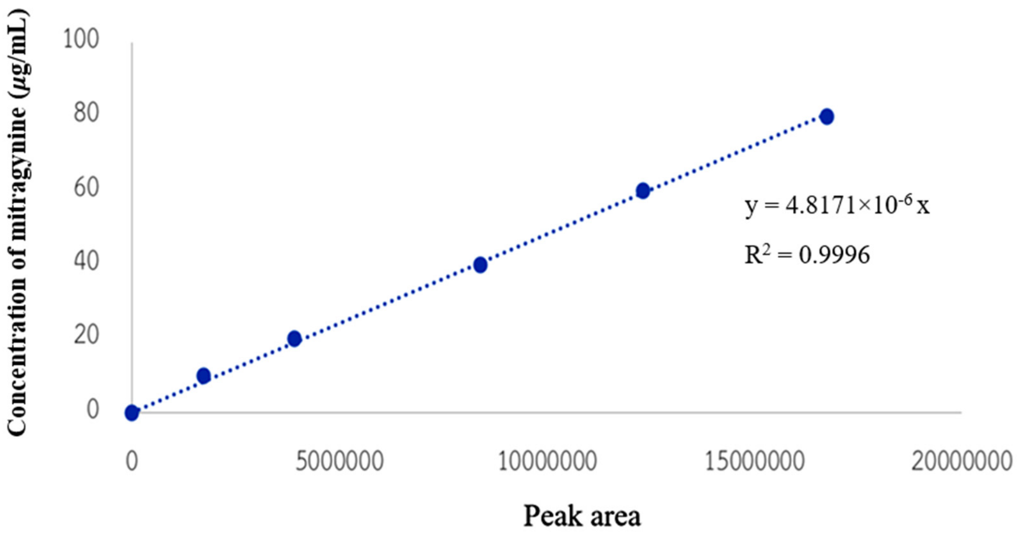

Figure S2. A standard curve of concentration (y-axis) vs. peak area (x-axis) made from standard samples of mitragynine for a calibration method to quantify unknown samples.

Table S1. Biological compatibility and cytocompatibility of kratom/mitragynine-based systems.

Study	Material/System	Cell Line(s)	Relation to present study
Wungsintaweekul et al., 2026 [15]	Kratom-loaded polymer film patch	Ex vivo skin; rabbit irritation model	Demonstrates that kratom-loaded polymer films are noncytotoxic and well tolerated on skin, supporting biomaterial-based delivery of kratom extract as a safe topical approach
Tuntiyasawasdikul et al., 2024 [14]	*M. speciosa* ethanolic extract	Human skin cells	Confirms that the ethanolic extract remains noncytotoxic within a defined concentration window and is stable upon formulation
Goh et al., 2021 [47]	Accelerated solvent extraction (ASE) kratom extract	Fibroblast, melanocyte, macrophage, keratinocyte	Establishes cytocompatibility of kratom extract across multiple skin- and immune-relevant cell types, providing a reference concentration range for noncytotoxic use
Anantaworasakul et al., 2025 [51]	Kratom nanoparticle formulations	HaCaT keratinocytes	Shows that keratinocyte viability remains high across kratom nanoparticle formulations, supporting the cytocompatibility of kratom-based delivery systems on skin cells
Syed et al., 2023 [24]	*M. speciosa* extract-loaded nanostructured lipid carriers (NLC)	Formulation/preformulation (cell studies pending)	Represents an alternative biomaterial platform explored for kratom delivery; no bacterial-cellulose-based system has previously been reported
Thepthong et al., 2025 [24]	Chitosan-encapsulated *M. speciosa* alkaloids	Formulation-level (cell studies pending)	Illustrates active exploration of biomaterial matrices for kratom alkaloid delivery, none of which has used bacterial cellulose to date

Table S2. Antioxidant activities reported for kratom.

Study	Compound	Assay	Relation to present study
Kaewchompoo et al., 2025 [52]	Kratom ethanolic (Et-MS) and alkaloid-rich (Alk-MS) extracts, purified mitragynine	DPPH, ABTS, FRAP	Demonstrates strong radical-scavenging activity of ethanolic kratom extract, supporting its use as a source of antioxidant bioactivity
Rahmawati et al., 2024 [42]	Kratom alkaloid extract (~46% mitragynine)	ROS scavenging in LPS/H2O2-stimulated RAW264.7	Shows significant reduction of intracellular ROS/RNS without cytotoxicity, providing a mechanistic basis for the antioxidant properties of kratom alkaloid extract
Anantaworasakul et al., 2025 [51]	Kratom NP formulations (4 varieties)	DPPH, ABTS, FRAP, ROS scavenging (cell-based)	Confirms that antioxidant activity is retained after nanoparticle formulation, supporting the feasibility of preserving antioxidant activity within a delivery matrix

Table S3. Anti-inflammatory activities reported for kratom/mitragynine.

Study	Compound	Markers Assessed	Relation to present study
Tuntiyasawasdikul et al., 2024 [14]	*M. speciosa* ethanolic extract	NO, pro-inflammatory cytokines	Demonstrates dose-dependent suppression of NO and pro-inflammatory cytokine expression, reinforcing the anti-inflammatory basis of kratom ethanolic extract
Rahmawati et al., 2024 [42]	Kratom alkaloid extract (~46% mitragynine) vs. crude extract (~5%)	TNF-α, IL-6, COX-2, 5-LOX, ROS	Shows that alkaloid-rich extract markedly reduces TNF-α/IL-6 and inhibits COX-2/5-LOX without cytotoxicity, directly supporting the anti-inflammatory potential attributed to mitragynine
Kafo et al., 2023 [49]	*M. speciosa* methanolic extract (MSME)	IL-6, TNF-α, IL-1β, ROS, phagocytosis	Demonstrates downregulation of pro-inflammatory cytokine expression and macrophage phagocytic activity, supporting cytokine-level anti-inflammatory evidence for kratom extract
Salim et al., 2022 [41]	*M. speciosa* methanol extract (75–200 mg/kg)	iNOS, COX-2, NF-κB(p65), ERK/JNK (MAPK)	Demonstrates dose-dependent suppression of iNOS/COX-2 expression and NF-κB nuclear translocation in vivo, supporting the anti-inflammatory mechanism attributed to kratom extract

Table S4. Evidence supporting wound-healing potential of kratom and mitragynine.

Study	Sample	Model	Relation to present study
Wungsintaweekul et al., 2026 [15]	Kratom extract-loaded polymer film patch (agar/pectin/alginate/PVA)	Ex vivo pig-skin permeation; rabbit skin irritation screening	Demonstrates absence of skin irritation and controlled permeation, supporting the feasibility of film-based topical/transdermal kratom delivery as a noncytotoxic wound-dressing platform
Rahmawati et al., 2024 [42]	Kratom alkaloid extract (~46% mitragynine)	LPS-induced RAW264.7; ROS/NO/COX-2/5-LOX assays	Shows suppression of ROS/NO and inflammatory mediators relevant to the inflammatory phase of wound healing, providing mechanistic rationale for wound-healing-supportive potential
Zakaria et al., 2023 [50]	*M. speciosa* methanol crude extract + DCM/EtOAc fractions	3T3 fibroblast & HUVEC; scratch/migration assay, tube-formation (angiogenesis) assay	Demonstrates enhanced fibroblast/endothelial migration and angiogenic activity at non-toxic concentrations, directly supporting the biological plausibility of kratom extract as a wound-healing-relevant agent
